# Relationship between site of myocardial infarction, left ventricular function and cytokine levels in patients undergoing coronary artery surgery

**DOI:** 10.5830/CVJA-2016-027

**Published:** 2016

**Authors:** Kiris Ilker, Kapan Sahin, Okutan, Huseyin, Narin Cuneyt, Ozaydın Mehmet, Cumhur Cure Medine, Sutcu Recep

**Affiliations:** Department of Cardiovascular Surgery, Medifema Private Hospital, Izmir, Turkey; Department of Cardiovascular Surgery, Medical Park Antalya Hospital, Antalya, Turkey; Department of Cardiovascular Surgery, Medical Park Antalya Hospital, Antalya, Turkey; Department of Cardiovascular Surgery, Egepol Private Hospital, Izmir, Turkey; Department of Cardiology, Suleyman Demirel University Medical School, Isparta, Turkey; Department of Biochemistry, Recep Tayyip Erdogan University Medical School, Rize, Turkey; Department of Biochemistry, Ataturk Education and Research Hospital, Katip Celebi University, Izmir, Turkey

**Keywords:** cytokine,, left ventricle, myocardial infarction, coronary artery bypass grafting, pericardium, plasma

## Abstract

**Background:**

The purpose of this study was to examine the relationship between left ventricular (LV) function, cytokine levels and site of myocardial infarction (MI) in patients undergoing coronary artery bypass grafting (CABG).

**Methods:**

Sixty patients undergoing CABG were divided into three groups (n = 20) according to their history of site of myocardial infarction (MI): no previous MI, anterior MI and posterior/inferior MI. In the pre-operative period, detailed analysis of LV function was done by transthoracic echocardiography.The levels of adrenomedullin, interleukin-1-beta,interleukin-6, tumour necrosis factor-alpha (TNF-α) and angiotensin-II in both peripheral blood samples and pericardial fluid were also measured.

**Results:**

Echocardiographic analyses showed that the anterior MI group had significantly worse LV function than both the group with no previous MI and the posterior/inferior MI group (p < 0.05 for LV end-systolic diameter, fractional shortening, LV end-systolic volume, LV end-systolic volume index and ejection fraction). In the anterior MI group, both plasma and pericardial fluid levels of adrenomedullin and and pericardial fluid levels of interleukin-6 and interleukin- 1-beta were significantly higher than those in the group with no previous MI (p < 0.05), and pericardial fluid levels of adrenomedullin, interleukin-6 and interleukin-1-beta were significantly higher than those in the posterior/inferior MI group (p < 0.05).

**Conclusions:**

The results of this study indicate that (1) patients with an anterior MI had worse LV function than patients with no previous MI and those with a posterior/inferior MI, and (2) cytokine levels in the plasma and pericardial fluid in patients with anterior MI were increased compared to patients with no previous MI.

## Background

Transmural myocardial infarction (MI) results in neurohormonal activation as a compensation for the impaired contractile force of the myocardium.^1^ This neurohormonal activation is known to result in the synthesis and release of several cytokines and growth factors by the injured myocardium into the circulation.

Plasma levels of tumour necrosis factor-alpha (TNF-α) and interleukin-6 (IL-6) have been found to increase in patients with left ventricular (LV) dysfunction as their functional heart failure classification deteriorates.^2^ Serum concentrations of pro-inflammatory cytokines such as interleukin-1-beta (IL-1β),IL-6 and high-sensitivity C reactive protein were reported to be significantly elevated in patients with non-ST elevation acute coronary syndrome in whom new coronary events developed.^3^

Serneri et al.^4^ reported that the clinical course of heart failure is associated with a progressive increase in formation of cardiac angiotensin-II. Yoshitomi et al. reported that plasma adrenomedullin increased in the early phases of acute MI and was further elevated in patients with congestive heart failure.^5^ All these findings reveal a possible relationship between circulating levels of pro-inflammatory cytokines and LV function after acute MI.

In addition to increased cytokine levels in the circulation after acute MI and congestive heart failure, the injured myocardium may also produce cytokines locally and subsequently release them into the pericardial fluid. Since the layers of pericardium are lined with mesothelial cells, derived from the same stem cells as vascular endothelial cells, it is speculated that these cells may also synthesise and release vasoactive substances into the pericardial fluid.^6^ The cytokines in pericardial fluid may reflect the extent of coronary atherosclerosis and may also directly promote the atherosclerotic process.

Consistent with this hypothesis, the level of IL-1β in pericardial fluid in patients with ischaemic heart disease was found to behigher than in patients with both valvular and congenital heart disease.^7^ Namiki et al.^8^ reported that endothelin-1 concentrations in the pericardial fluid were more elevated in patients with ischaemic heart disease than in those with non-ischaemic heart disease. In addition, Ege et al.^9^ reported that levels of IL-2R, IL-6, IL-8 and TNF-α in pericardial fluid were significantly higher than in the serum in patients with MI. The pericardial fluid is partially formed from cardiac interstitial fluid,which migrates through the epicardium,^10^ therefore vasoactive substances released into the myocardial interstitium may appear in the pericardial fluid.^6^

Although levels of pro-inflammatory cytokines are well documented in both MI and heart failure, the relationship between cytokine levels, LV function and location of MI has not been fully clarified. The purpose of this study was to examine the relationship between LV function, cytokine levels and site of MI in patients undergoing coronary artery bypass grafting (CABG).

For this purpose, the patients undergoing CABG were divided into three groups according to the history of site of MI: anterior MI, posterior/inferior MI and no previous MI. LV function was analysed by transthoracic echocardiography and the levels of adrenomedullin, TNF-α, IL-1β, IL-6 and angiotensin-II in both the plasma and pericardial fluid were measured in these subgroups of patients.

## Methods

From September 2006 to September 2007, 60 patients who underwent primary CABG surgery were enrolled in this prospective study. There were 54 (90%) males and the mean age of the patients was 60.89 ± 9.39 years.

Coronary angiography and 12-lead electrocardiograms (ECG) were performed on each patient. All patients had documented coronary artery disease, defined as more than 75% stenosis in one or more of the principal coronary arteries, determined by coronary angiography.

Patients who had a recent MI in the last three weeks,emergent operation, coronary artery re-operation, cardiogenic shock, complications of acute MI (LV aneurysm, post-infarction ventricular septal defect or free wall rupture), haemodynamically significant valvular disease (severe regurgitation of more than two degrees or severe stenosis requiring surgical intervention),atrial fibrillation, active infectious disease, malignancies, chronic inflammatory disease or renal dysfunction were excluded from the study.

The baseline characteristics of the patients are shown in Table 1. Ongoing drug treatment included beta-blockers, angiotensin converting enzyme inhibitors, nitrates, calcium channel blockers and diuretics. All drugs were withheld on the day of the study

**Table 1:** Baseline characteristics of the patients

**	*No previous*	**	*Posterior/*	**
**	*MI*	*Anterior MI*	*inferior MI*	**
*Parameters*	*(n = 20)*	*(n = 20)*	*(n = 20)*	*p-value*
Male/female	18/2	18/2	18/2	1.00
Age (years)	57.65 ± 8.15	63.26 ± 9.90	61.90 ± 9.60	0.097
Body mass index (kg/m^2^)	26.03 ± 8.31	27.10 ± 7.86	26.81 ± 9.02	0.842
Drugs				
Beta-blockers	8	9	4	0.054
ACE inhibitors	8	7	4	0.061
Nitrates	20	20	20	1.00
Calcium channel blockers	5	2	3	0.062
Hypertension	11	11	5	0.089
Hyperlipidaemia	13	9	12	0.414
Smoking	13	12	17	0.189
Diabetes mellitus	4	7	3	0.298
COPD	2	2	3	0.851
Peripheral artery disease	1	1	1	1.00
Cerebrovascular event	0	0	0	1.00
Coronary artery stenting	1	0	1	0.596
Left main coronary artery disease	3	1	2	0.574

COPD = chronic obstructive pulmonary disease.

According to the ECG and cardiac catheterisation findings,patients were divided into three groups.^1^ The group with no previous MI (n = 20) included patients with no documented history of transmural MI. The anterior MI group (n = 20) included patients who had a total occlusion in the left anterior descending (LAD) coronary artery or q-waves in at least two anterior ECG leads. The posterior/inferior MI group (n = 20) included patients who had a total occlusion in the right coronary artery (RCA) or left circumflex coronary artery (LCx), or q-waves in the posterior–inferior ECG leads.

This study was conducted in accordance with guidelines approved by the ethics committee at our institution. Informed consent was obtained from each participant prior to inclusion in the study.

Standard anaesthesia and anaesthetic techniques were used in all patients by the same anaesthesiology team. Following a median sternotomy, the ascending aorta was cannulated for arterial inflow and the right atrial appendage was cannulated with a two-stage cannula for venous uptake. A cardioplegic tack was introduced into the aortic root, proximal to the aortic cannulation site for antegrade cardioplegic delivery. Heparin was given at a dose of 3 mg/kg for systemic anticoagulation, and cardiopulmonary bypass was established.

Myocardial protection was maintained initially using cold (0–4°C) crystalloid cardioplegia solution, followed by cold blood (10ºC) cardioplegia, and finally warm blood (37°C) cardioplegia. Mild systemic hypothermia (32°C) was applied.

We used the left and right internal thoracic arteries and the radial artery as arterial grafts, and the saphenous vein as venous graft during CABG. If the left internal thoracic artery was in optimal condition and had pulsatile flow, it was preferentially anastomosed to the left anterior descending coronary artery.

After a median sternotomy, the mediastinal adipose tissue and thymus were displaced from the pericardium, which was opened and pericardial fluid was collected. Contact between the pericardial fluid and blood was meticulously avoided.

Arterial blood samples were simultaneously withdrawn from an intra-arterial cannula. The samples were immediately transferred into glass tubes and centrifuged at 3 500 rpm for four minutes. The samples were kept at –80°C for subsequent assays.

Levels of adrenomedullin, IL-6, TNF-α, IL-1β and angiotensin-II in the plasma and pericardial fluid were measured. Adrenomedullin levels were measured with a commercial kit (Phoenix Pharmaceuticals Inc, CA, USA) using the enzyme immunoassay (EIA) method. IL-6, TNF-α and IL-1β levels were measured with commercial kits (Biosource Diagnostics, Nivelles, Belgium) using the EIA method. Angiotensin-II levels were measured with a commercial kit (Biosource Diagnostics, Nivelles, Belgium) and radioimmunoassay (RIA) method.

LV function was analysed in detail in all patients pre-operatively by transthoracic echocardiography (Vingmed System 5 Performance™, General Electric, USA). Measured indices of LV function were LV end-diastolic diameter (LVEDD), LV end-diastolic volume (LVEDV), LV end-diastolic volume index (LVEDVI), LV end-systolic diameter (LVESD), LV end-systolic volume (LVESV), LV end-systolic volume index (LVESVI), fractional shortening (FS) and LV ejection fraction (LVEF). A two-dimensional echocardiogram from the apical view was used for determination of LVEF by single-plane planimetry of the left ventricle (modified Simpson method).

## Statistical analysis

For continuous variables, results are presented as mean ± standard deviation (SD). As the values obtained were not normally distributed, non-parametric methods were used for tests of significance. The Kruskall–Wallis test was used to compare the means between the three groups (no previous MI, anterior MI and posterior/inferior MI). If this test indicated a significant difference between the groups, the Mann–Whitney U-test was used to compare differences between the groups.

Categorical variables are presented by frequency counts, and differences between the groups with regard to categorised data were compared with the chi-squared test. All calculations were performed using a standard statistical package (SPSS 15.0, SPSS Inc, Chicago, IL, USA). All p-values < 0.05 were interpreted as statistically significant.

## Results

The groups were homogenous for baseline characteristics in the pre-operative period (p > 0.05 for all comparisons) (Table 1). Details of the surgery performed and the early postoperative period is shown in Table 2.

**Table 2:** Details of surgery and the early postoperative period

**	*No previous*	**	*Posterior/*	**
**	*MI*	*Anterior MI*	*inferior MI*	**
**	*(n = 20)*	*(n = 20)*	*(n = 20)*	*p-value*
Number of distal anastomoses	2.62 ± 0.80	2.54 ± 0.82	2.66 ± 0.48	0.136
CPB time (min)	99.25 ± 25.08	94.81 ± 25.47	111.44 ± 33.59	0.254
ACC time (min)	55.93 ± 16.57	51.81 ± 15.08	57.11 ± 16.57	0.740
Extubation time (h)	6.88 ± 1.48	9.11 ± 8.29	8.02 ± 4.79	0.642
Stay in intensive care unit (days)	2.00 ± 0.00	2.11 ± 0.32	2.35 ± 0.87	0.190
Positive inotropic drugs	3	9	9	0.072
Intra-aortic balloon pump	1	2	1	0.765
Acute renal failure	–	1	–	0.437
Exitus	–	1	1	0.382

CPB = cardiopulmonary bypass, ACC = aortic cross-clamping time.

For the whole group of patients, mean number of distal anastomoses was 2.45 ± 0.81, cardiopulmonary bypass time was 101.77 ± 28.71 min, and aortic cross-clamping time was 54.93 ± 16.19 min. The mean extubation time and length of stay in the intensive care unit were 7.98 ± 5.52 hours and 2.15 ± 0.56 days, respectively.

Off-pump CABG was performed in one patient in the group with no previous MI, in four patients in the anterior MI group and in two patients in the posterior/inferior MI group. Two patients in the anterior MI group and one patient in the posterior/inferior MI group underwent re-exploration due to excessive mediastinal bleeding in the early postoperative period.

The left internal thoracic artery (ITA) was anastomosed to the LAD in 57 patients and a saphenous vein graft was used for the remaining patients. The right ITA was used as a graft in four patients in the group with no previous MI, and in two patients in the posterior/inferior MI group. The radial artery was used as a graft in two patients in the posterior/inferior group.

Positive inotropic support was used in 21 patients, and intra-aortic balloon pump was required in four in the early postoperative period. There were two in-hospital deaths. The patient in the anterior MI group died due to acute renal failure. The patient in the posterior/inferior MI group died due to low cardiac output and multiple organ failure.

There were no statistically significant differences between the groups in terms of parameters of the intra-operative and early postoperative periods (p > 0.05 for mean number of distal anastomosis, cardiopulmonary bypass time, aortic crossclamping time, extubation time, length of stay in intensive care unit, use of positive inotropic support, insertion of intra-aortic balloon pump, incidence of acute renal failure and mortality).

**Fig .1:**
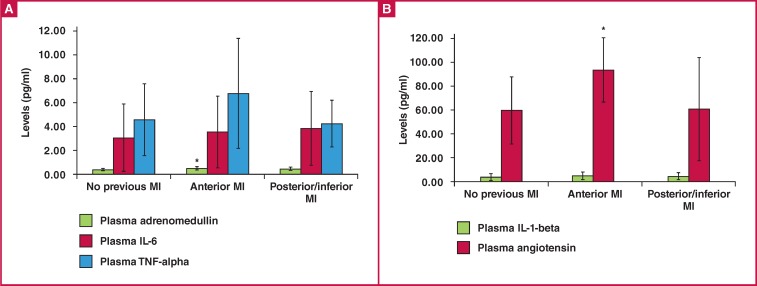
A. The levels of adrenomedullin, IL-6 and TNF-alpha in plasma. *p & 0.05 for adrenomedullin in the anterior MI group vs in the other groups. B. The levels of IL-1-beta and angiotensin-II in plasma. *p & 0.05 for angiotensin in the anterior MI group vs in the group with no previous MI. IL-6 = interleukin-6, TNF-alpha = tumour necrosis factor-alpha, IL-1-beta = interleukin- 1-beta, Angiotensin = angiotensin-II.

Levels of adrenomedullin, IL-6 and TNF-α in the plasma are shown in Fig. 1A. Levels of IL-1β and angiotensin-II in the plasma are shown in Fig. 1B. The plasma level of adrenomedullin in the anterior MI group (0.42 ± 0.15 ng/ml) was significantly higher than that in the group with no previous MI (0.30 ± 0.07 ng/ml) and the posterior/inferior MI group (0.33 ± 0.05 ng/ml) (p = 0.002 and p = 0.043, respectively) (Fig. 1A).

There were no statistically significant difference between the plasma levels of IL-6 in the group with no previous MI, the anterior MI and posterior/inferior MI groups (3.14 ± 2.84, 3.62 ± 2.93 and 3.53 ± 2.91 pg/ml, p = 0.414) (Fig. 1A). There were no statistically significant difference between the plasma levels of TNF-α in the group with no previous MI, the anterior MI and the posterior/inferior MI groups (4.48 ± 2.93, 6.63 ± 4.41 and 4.38 ± 1.78 pg/ml, p = 0.322) (Fig. 1A).

There were no statistically significant differences between the plasma levels of IL-1β in the group with no previous MI, the anterior MI and the posterior/inferior MI groups (4.15 ± 2.64, 4.62 ± 3.83 and 4.46 ± 2.86 pg/ml, p = 0.977) (Fig. 1B).

The plasma level of angiotensin-II in the anterior MI group was significantly higher than that in the group with no previous MI (91.30 ± 26.40 vs 60.80 ± 27.94 pmol/l, p = 0.002) (Fig. 1B).

**Fig .2:**
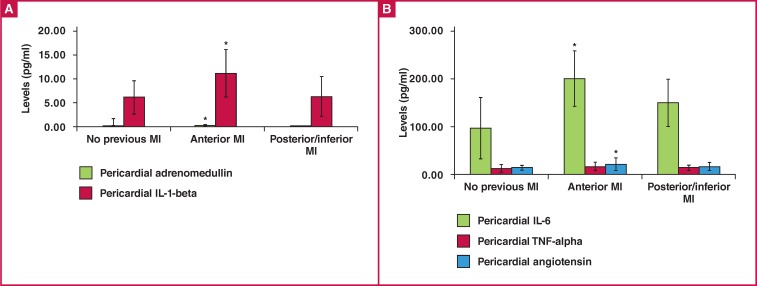
A. The levels of adrenomedullin and IL-1-beta in pericardial fluid. *p < 0.05 for adrenomedullin in the anterior MI group vs in the group with no previous MI, †p < 0.05 for IL-1-beta in the anterior MI group vs in the other groups. B. The levels of IL-6, TNF-alpha and angiotensin-II in pericardial fluid. *p < 0.05 for IL-6 in the anterior MI group vs in the other groups, †p < 0.05 for angiotensin-II in the anterior MI group vs in the group with no previous MI. TNF-alpha = tumour necrosis factor-alpha, IL-1-beta = interleukin-1-beta, Angiotensin = angiotensin-II.

Levels of adrenomedullin and IL-1β in the pericardial fluid are shown in Fig. 2A. Levels of IL-6, TNF-α and angiotensin- II in the pericardial fluid are shown in Fig. 2B. The level of adrenomedullin in the pericardial fluid in the anterior MI group was significantly higher than that in the group with no previous MI (0.52 ± 0.14 vs 0.42 ± 0.08 ng/ml, p = 0.028) (Fig. 2A).

The level of IL-1β in the pericardial fluid in the anterior MI group (10.54 ± 5.17 pg/ml) was significantly higher than that in both the group with no previous MI (5.96 ± 3.68 pg/ml) and the posterior/inferior MI group (6.08 ± 4.10 pg/ml) (p = 0.008 and p = 0.005, respectively) (Fig. 2A).

The level of IL-6 in the pericardial fluid in the anterior MI group (193.51 ± 62.29 pg/ml) was significantly higher than that in both the group with no previous MI (105.25 ± 69.71 pg/ml) and the posterior/inferior MI group (139.91 ± 54.18 pg/ml) (p = 0.000and p = 0.033, respectively) (Fig. 2B). There were no statistically significant differences between the levels of TNF-α in the pericardial fluid in the group with no previous MI, the anterior MI and the posterior/inferior MI groups (13.08 ± 8.66, 19.01 ± 10.37 and 14.99 ± 6.85 pg/ml, p = 0.203) (Fig. 2B). The level of angiotensin-II in the pericardial fluid in the anterior MI group was significantly higher than that in the group with no previous MI (21.83 ± 14.48 vs 14.60 ± 5.25 pmol/l, p = 0.019) (Fig. 2B).

**Fig .3:**
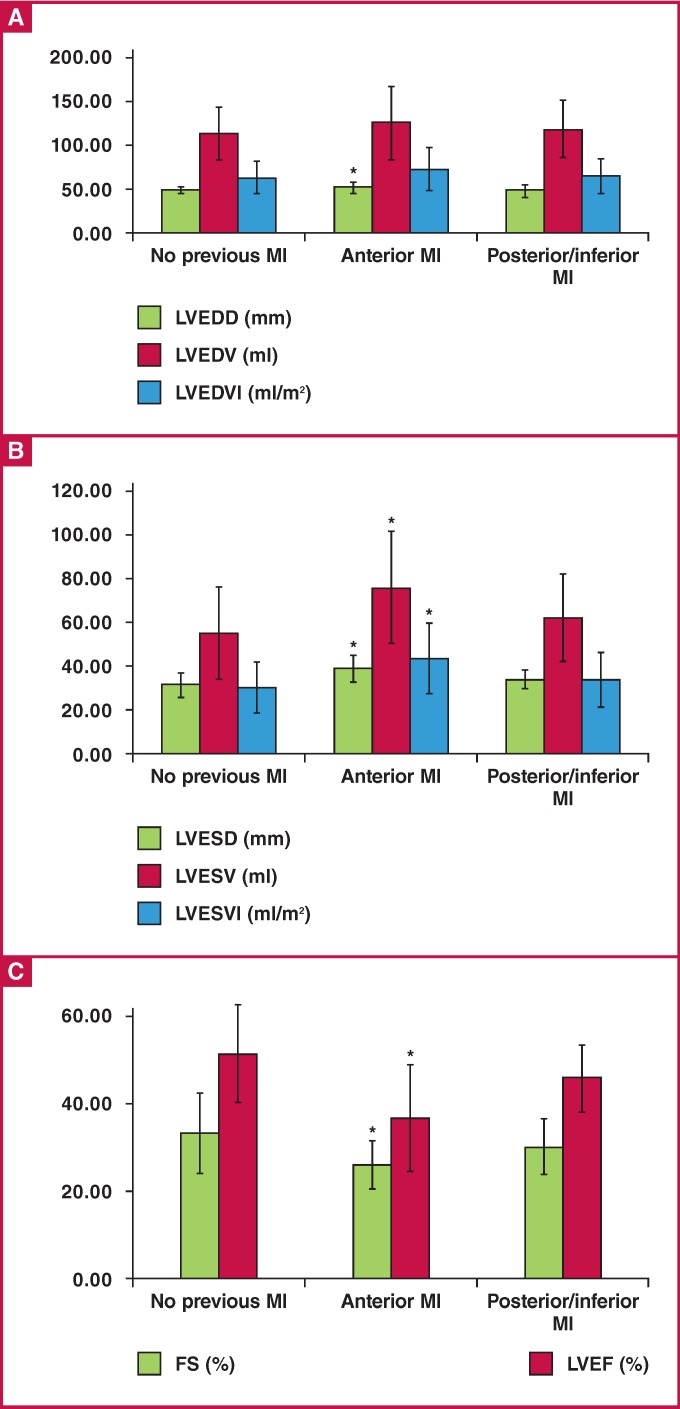
Results of echocardiographic analyses. A. *p < 0.05 for LVEDD in the anterior MI group vs in the group with no previous MI. B. *p < 0.05 for LVESD, LVESV and LVESVI in the anterior MI group vs in the other groups. C. *p < 0.05 for FS and LVEF in the anterior MI group vs in the other groups. LVEDD = left ventricular enddiastolic diameter, LVEDV = left ventricular end-diastolic volume, LVEDVI = left ventricular end-diastolic volume index, LVESD = left ventricular end-systolic diameter, LVESV = left ventricular end-systolic volume, LVESVI = left ventricular end-systolic volume index, FS = fractional shortening, LVEF = left ventricular ejection fraction.

The results of LVEDD, LVEDV and LVEDVI in the three groups are shown in Fig. 3A. The results of LVESD, LVESV and LVESVI in the groups are shown in Fig. 3B. The results of FS (%) and EF (%) in the groups are shown in Fig. 3C

The mean LVEDD in the anterior MI group was significantly higher than that in the group with no previous MI (53.39 ± 4.43 vs 49.05 ± 3.79 mm, p = 0.004) (Fig. 3A). There were no statistically significant differences between the mean LVEDV in the group with no previous MI, the anterior MI and the posterior/inferior MI groups (113.97 ± 31.13, 127.14 ± 39.19 and 118.20 ± 33.55 ml, respectively, p = 0.474) (Fig. 3A). There were no statistically significant differences between the mean LVEDVI in the group with no previous MI, the anterior MI and the posterior/inferior MI groups (63.22 ± 18.58, 73.03 ± 24.34 and 65.57 ± 18.54 ml/m2, p = 0.366) (Fig. 3A).

The mean LVESD in the anterior MI group (39.23 ± 5.46 mm) was significantly higher than that in both the group with no previous MI (32.10 ± 5.25 mm) and the posterior/inferior MI group (33.75 ± 4.54 mm) (p = 0.000 and p = 0.003, respectively) (Fig. 3B). The mean LVESV in the anterior MI group (76.29 ± 25.95 ml) was significantly higher than that in both the group with no previous MI (55.24 ± 21.76 ml) and the posterior/inferior MI group (62.35 ± 19.86 ml) (p = 0.002 and p = 0.028, respectively) (Fig. 3B). The mean LVESVI in the anterior MI group (43.74 ± 16.11 ml/m2) was significantly higher than that in both the group with no previous MI (30.52 ± 12.02 ml/m2) and the posterior/inferior MI group (34.67 ± 11.76 ml/m2) (p = 0.002 and p = 0.026, respectively) (Fig. 3B).

The mean LVEF in the anterior MI group (36.90 ± 12.21%) was significantly lower than that in both the group with no previous MI (51.62 ± 10.97%) and the posterior/inferior MI group (46.00 ± 7.54%) (p = 0.002 and p = 0.024, respectively) (Fig. 3C). The differences in FS values between the groups were similar to the differences in EF values (Fig. 3C).

## Discussion

The results of this study indicate that (1) the patients who had suffered an anterior MI had worse LV function than both those with no previous MI and those with posterior/inferior MI, and (2) the levels of pro-inflammatory cytokines in the plasma and pericardial fluid in patients with anterior MI were increased compared to patients with no previous MI.

Adrenomedullin, a 52-amino acid peptide with structural homology to calcitonin gene-related peptide, was initially isolated from human phaeochromocytoma.^11^ Adrenomedullin is synthesised by many mammalian tissues, including the adrenal medulla, endothelial and vascular smooth muscle cells, myocardium and central nervous system.^12^

Clinical studies suggest that synthesis of adrenomedullin is up-regulated during myocardial ischaemia. Measurement of plasma levels of adrenomedullin in patients in the acute stages of MI showed elevated circulating levels of adrenomedullin within 24 to 48 hours of admission, which gradually decreased over a three-week period.^13^ On the other hand, Miyao et al.^14^ reported that in patients with acute MI, increased plasma levels of adrenomedullin in the very early phase of acute MI returned to normal limits approximately four weeks later.

In our study, the timespan between MI and CABG was three weeks or longer. We found that plasma adrenomedullin levels in both the anterior MI and the posterior/inferior MI groups were higher than that in the group with no previous MI. In agreement with the results of Miyao et al.,^14^ our results suggest that the elevated adrenomedullin levels were most likely a consequence of the recent MI.

It is generally considered that pericardial fluid is not merely an ultra-filtrate of plasma, but also a transudate from the cardiac interstitium.^15^ Adrenomedullin mRNA is expressed by several cardiovascular tissues, including the cardiomyocytes, vascular endothelial and smooth muscle cells.^12^ Therefore, it can be assumed that the level of adrenomedullin in pericardial fluid may increase concomitantly with plasma levels.

Supporting this assumption, increased pericardial fluid concentrations of adrenomedullin have been reported in patients with cardiac remodelling.^16^ Additionally, adrenomedullin levels were reported to be slightly higher in the pericardial fluid than in the plasma in patients undergoing CABG.^17^ Consistent with this report, we also found that adrenomedullin levels in the pericardial fluid were slightly higher than those in the plasma in all three groups.

In our study, the anterior MI group had the worst LV function, as shown by echocardiography. Miyao et al. suggested that adrenomedullin levels in patients with acute MI may indirectly reflect the extent of ventricular dysfunction.^14^ In addition,mechanical stretch, angiotensin-II and pro-inflammatory cytokines synthesised in the infarcted area may also stimulate adrenomedullin production.^12^

Activation of the renin–angiotensin–aldosterone system commonly accompanies MI.^18^ Angiotensin-II, a potent vasoconstrictor, is involved with vascular tone and endothelial function, cardiac contractility, impulse propagation, and it stimulates the formation and secretion of aldosterone from the adrenal gland.^19^ We found increased levels of angiotensin-II in both the plasma and pericardial fluid in the anterior MI group.

Schunkert et al.20 reported that plasma angiotensin-II levels were increased six weeks after experimental MI in rats with congestive heart failure. Both our finding and the results reported by Schunkert et al.^20^ suggest activation of the renin–angiotensin system and a subsequent increase in circulating angiotensin-II.

On the other hand, Huang et al.^21^ found that in rats three months after subjection to MI, the plasma renin level was increased but plasma angiotensin-II levels were not different from those in the control group. The authors concluded that decreased lung angiotensin converting enzyme activity could possibly have contributed to keeping plasma angiotensin-II levels in the normal range. Another explanation may be the clearance of angiotensin-II from the circulation in the three-month period after MI.

Serneri et al.^4^ found that the clinical course of heart failure is associated with a progressive increase in cardiac angiotensin- II formation, as expressed by the mean aorta–coronary sinus concentration gradient. In agreement with this study, we found the highest pericardial fluid angiotensin-II level was in the anterior MI group, the group which had the worst LV function.

IL-6 is a classic multifunctional cytokine, with several activities that could explain its potential importance in acute coronary syndromes.^22^ In addition, IL-6 has been suggested as a marker of severity of coronary artery disease, since increased plasma concentrations and activated myocardial gene expression have been demonstrated after MI.

IL-1 is a prototypic pro-inflammatory cytokine with a wide range of actions systemically and at the cardiovascular level.^23^ The IL-1 family encompasses IL-1α, IL-1β and IL-1Ra and is mainly produced by monocytes and macrophages, and to a lesser degree by endothelial cells.^23^

Birner et al.^24^ performed a human study in which plasma N-terminal proBNP (NT-proBNP) and IL-6 levels were measured in a large group of patients in the chronic phase after MI and found that both NT-proBNP and IL-6 levels were significantly elevated in subjects with MI compared to the control group. When they analysed NT-proBNP and IL-6 levels with regard to EF, they observed a significant increase in NT-proBNP levels in the presence of LV dysfunction. By contrast, IL-6 level did not increase further in MI subjects with LV dysfunction, compared to MI subjects with preserved LV function. These findings may suggest that plasma levels of IL-6 are not as sensitive as NT-proBNP as a biomarker of LV dysfunction in the presence of MI.

We also found that the levels of IL-6 and IL-1β in plasma did not differ significantly between the groups. The lack of significant elevation of plasma levels of IL-6 in patients with MI in our study could have been due to insufficient numbers of patients. These findings could also be interpreted that levels of IL-6 and IL-1β in plasma were not influenced by the site of MI.

On the other hand, the elapsed time from MI seems to be an important factor in the marker role of IL-6 and IL-1β on MI. In addition, IL-1α and IL-1β lack a signal peptide and they are not readily secreted into the systemic circulation and therefore determination of plasma level is unreliable.^25^

Plasma levels of IL-1Ra, a sensitive marker of biologically active IL-1β, and IL-6 were measured at the time of admission to the coronary care unit and 48 hours later in patients who were hospitalised due to unstable angina.^25^ The authors found that a fall in IL-1Ra and IL-6 48 hours after admission was associated with an uneventful course.

In our study, complicated medical conditions such as development of new cardiac events, emergent operation, cardiogenic shock or complications of acute MI were all exclusion criteria. Therefore the lack of difference in plasma levels of IL-6 and IL-1β in our study could also be partly due to their relatively stable medical status and the absence of major new-onset cardiac events.

We also found that pericardial fluid levels of IL-1β and IL-6 were markedly increased in the anterior MI group. This indicates that pericardial fluid levels of these two cytokines may be superior to plasma levels as a marker of LV dysfunction in the setting of MI. It has also been reported that pericardial concentrations of IL-1β may reflect the extent of ischaemic heart disease and that elevated IL-1β concentrations in pericardial fluid may also directly promote the process of coronary atherosclerosis.^7^

TNF-α is a multifunctional circulating cytokine derived from endothelial and smooth muscle cells as well as macrophages associated with coronary atheroma.^26^,^27^ TNF-α possesses cytotoxic and negative inotropic actions, aggravates the inflammatory process, and plays a role in neutrophil pre-activation and ischaemic injury.^28^,^29^ Brunetti et al.^30^ reported that levels of TNF-α in patients with acute coronary syndrome were associated with a worse prognosis at follow up. Prior data have demonstrated that those individuals with evidence of severely reduced ejection fraction and clinical heart failure had markedly elevated levels of TNF-α.^31^,^32^ Torre-Amione et al.^33^ also reported that concentrations of TNF-α were high in patients with heart failure, in association with noticeable activation of the renin– angiotensin system. There was, however, a wide variation in TNF-α levels between patients and in many it was not detected.

Dutka et al.^34^ examined the concentrations of circulating TNF-α in patients with congestive heart failure and found that the mean concentration of TNF-α was greater than the upper 95% confidence interval for healthy controls, but there was considerable between- and within-patient variation. Therefore the authors concluded that the stimulus resulting in enhanced plasma concentrations of TNF-α in congestive heart failure remains unclear and concentrations at any particular time were not prognostic.

In our study, although both plasma and pericardial fluid TNF-α levels in the anterior MI group were slightly higher than those in the other groups, the differences were not statistically significant. We believe that the lack of statistically significant elevation in the levels of TNF-α in the anterior MI group, the group which had the poorest LV function, may have been due to the absence of severe clinical heart failure. Another explanation could be that the wide variation in TNF-α levels between patients resulted in relatively high standard deviations and precluded finding significant differences with statistical analysis.

In our study, although LV function in the anterior MI group was significantly worse than that in the posterior/inferior MI group, only adrenomedullin level in the plasma and levels of IL-6 and IL-1β in pericardial fluid in the anterior MI group were significantly higher than those in the posterior/inferior MI group. Reviewing these findings, one may consider that there was a weak correlation between enhanced cytokine levels and depressed LV function. We believe these findings suggest that cytokine levels in the pericardial fluid may be superior to plasma levels as a molecular marker of LV dysfunction in the setting of MI. In addition, the number of patients included in our study may have been insufficient to observe a statistically significant difference in the levels of cytokines between the MI groups.

## Limitations

Our study was subject to certain limitations. First, we did not measure the extent of infarcted myocardial tissue by means of myocardial perfusion imaging techniques. Since the magnitude of MI may affect cytokine levels, one may consider that changes in cytokine levels may be partly attributed to the area of non-contractile myocardial tissue. However, we believe that a detailed assessment of LV function by echocardiography is sufficient to clarify the effect of MI on contractile myocardial tissue of the left ventricle. In addition, the aim of this study was to examine the relationship between MI site, cytokine levels and LV function. Therefore the relationship between the magnitude of MI and cytokine levels was beyond the scope of this study.

Second, in our study, the elapsed time between MI and CABG was three weeks or longer. This interval may be sufficient for an increase in certain cytokines, such as adrenomedullin and angiotensin-II, but too long for other cytokines, such as IL-6 or IL-1β, to remain high in the systemic circulation. The time points at which cytokines peak and the intervals in which cytokines remain high in the plasma differ. As Tashiro et al.^35^ stated, concentrations of monocyte-related cytokines dynamically change during the course of acute MI, suggesting that they may contribute to the inflammatory and subsequent proliferative responses in acute MI. Therefore levels in the pericardial fluid appear to be more reliable and superior to levels in the plasma as a molecular marker in the early stages of MI and LV dysfunction.

Third, the absence of a control group in our study is another limitation but pericardial fluid samples are obtained by pericardiocentesis or during cardiac surgery. Therefore obtaining pericardial fluid samples from healthy individuals was not possible, for ethical reasons.

## Conclusions

We found that (1) patients with anterior MI had worse LV function than both patients with no previous MI and those with posterior/inferior MI, and (2) the levels of pro-inflammatory cytokines in plasma and pericardial fluid in patients with anterior MI were increased compared to patients with no previous MI. The finding of elevated pro-inflammatory cytokine levels in patients with anterior MI could be interpreted as reflecting both the magnitude of MI and/or LV dysfunction and the site of MI

Our results also suggest that cytokine levels in pericardial fluid were superior to plasma levels as a molecular marker of LV dysfunction in the setting of MI. However, further clinical studies with larger patient numbers are required to clarify the prognostic or biomarker role of cytokines in pericardial fluid related to LV dysfunction or remodelling after acute MI.

The abstract of this study was presented in 57th International Congress of the European Society for Cardiovascular Surgery, Barcelona, Spain, 24–27 April 2008.

The study was financially supported by the Department of Scientific Research Projects, Suleyman Demirel University (project no: 1389-M-06). None of the authors has financial or other relationships that would influence assessment of the data or that would constitute a conflict of interest.

## References

[1] Klemola R, Tikkanen I, Vuolteenaho O, Toivonen L, Laine M (2001). Plasma and pericardial fluid natriuretic peptide levels in postinfarction ventricular dysfunction.. Eur J Heart Fail.

[2] Torre-Amione G, Kapadia S, Benedict C, Oral H, Young JB, Mann DL (1996). Proinflammatory cytokine levels in patients with depressed left ventricular ejection fraction: a report from the Studies of Left Ventricular Dysfunction (SOLVD).. J Am Coll Cardiol.

[3] Kilic T, Ural D, Yumuk Z (2006). Relation between proinflammatory to anti-inflammatory cytokine ratios and long-term prognosis in patients with non-ST elevation acute coronary syndrome.. Heart.

[4] Serneri GG, Boddi M, Cecioni I (2001). Cardiac angiotensin II formation in the clinical course of heart failure and its relationship with left ventricular function. Circ Res.

[5] Yoshitomi Y, Nishikimi T, Kojima S (1998). Plasma levels of adrenomedullin in patients with acute myocardial infarction. Clin Sci.

[6] Horkay F, Szokodi I, Selmeci L (1998). Presence of immunoreactive endothelin-1 and atrial natriuretic peptide in human pericardial fluid.. Life Sci.

[7] Oyama J, Shimokawa H, Morita S, Yasui H, Takeshita A (2001). Elevated interleukin-1beta in pericardial fluid of patients with ischemic heart disease.. Coron Artery Dis.

[8] Namiki A, Kubota T, Fukozawa M (2003). Endothelin-1 concentrations in pericardial fluid are more elevated in patients with ischemic heart disease than in patients with nonischemic heart disease.. Jpn Heart J.

[9] Ege T, Canbaz S, Yuksel V, Duran E (2003). Effect of pericardial fluid proinflammatory cytokines on hemodynamic parameters.. Cytokine.

[10] Gibson AT, Segal MB (1978). A study of composition of pericardial fluid, with special reference to the probable mechanism of fluid formation.. J Physiol.

[11] Kitamura K, Kangawa K, Kawamoto M (1993). Adrenomedullin: a novel hypotensive peptide isolated from human pheochromocytoma.. Biophys Res Commun.

[12] Beltowski J, Jamroz A (2004). Adrenomedullin – what do we know 10 years since its discovery?. Pol J Pharmacol.

[13] Kobayashi K, Kitamura K, Hirayama N (1996). Increased plasma adrenomedullin in acute myocardial infarction.. Am Heart J.

[14] Miyao Y, Nishikimi T, Goto Y (19998). Increased plasma adrenomedullin levels in patients with acute myocardial infarction in proportion to the clinical severity. Heart.

[15] Page E, Upshaw-Earley J, Goings G (1992). Permeability of rat atrial endocardium, epicardium, and myocardium to large molecules: stretchdependent effects. Circ Res.

[16] Tambara K, Fujita M, Nagaya N (2002). Increased pericardial fluid concentrations of the mature form of adrenomedullin in patients with cardiac remodeling.. Heart.

[17] Nishikimi T, Shibasaki I, Iıda H (2002). Molecular forms of adrenomedullin in pericardial fluid and plasma in patients with ischaemic heart disease.. Clin Sci.

[18] Anavekar NS, Solomon SD (2005). Angiotensin II receptor blockade and ventricular remodelling.. J Renin Angiotensin Aldosterone Syst.

[19] Timmermans P, Benfield P, Chiu AT (1992). Angiotensin II receptors and functional correlates.. Am J Hypertens.

[20] Schunkert H, Tang SS, Litwin SE (1993). Regulation of intrarenal and circulating rennin-angiotensin systems in severe heart failure in the rat.. Cardiovasc Res.

[21] Huang H, Arnal JF, Llorens-Cortes C (1994). Discrepancy between plasma and lung angiotensin-converting enzyme activity in experimental congestive heart failure. A novel aspect of endothelium dysfunction.. Circ Res.

[22] Simon AD, Yazdani S, Wang W, Schwartz A, Rabbani LE (2000). Circulating levels of IL-1β, a prothrombotic cytokine, are elevated in unstable angina versus stable angina.. J Thromb Thrombol.

[23] Debrunner M, Schuiki E, Minder E (2008). Proinflammatory cytokines in acute myocardial infraction with and without cardiogenic shock. Clin Res Cardiol.

[24] Birner CM, Ulucan C, Fredersdorf S (2007). Head-to-head comparison of BNP and IL-6 as markers of clinical and experimental heart failure: superiority of BNP.. Cytokine.

[25] Biasucci LM, Liuzzo G, Fantuzzi G (1999). Increasing levels of interleukin (IL)-1Ra and IL-6 during the first 2 days of hospitalization in unstable angina are associated with increased risk of in-hospital coronary events.. Circulation.

[26] Warner SJ, Libby P (1989). Human vascular smooth muscles: target for and source of tumor necrosis factor.. J Immunol.

[27] Tipping PG, Hancock WW (1994). Production of tumor necrosis factor and interleukin-1 by macrophages from human atheromatous plaques. Am J Pathol.

[28] Torre-Amione G, Kapadia S, Lee J (1996). Tumor necrosis factoralpha and tumor necrosis factor receptors in the failing human heart.. Circulation.

[29] Frenette PS (2001). Locking a leukocyte integrin with statins.. N Eng J Med.

[30] Brunetti ND, Munno I, Pellegrino PL, Ruggero V, Correale M, De Gennaro L (2011). Inflammatory cytokines imbalance in the very early phase of acute coronary syndrome: correlations with angiographic findings and in-hospital events.. Inflammation.

[31] Levine B, Kalman J, Mayer L, Fillit HM, Packer M (1990). Elevated circulating levels of tumor necrosis factor in severe chronic heart failure.. N Eng J Med.

[32] McMurray J, Abdullah I, Dargie H, Shapiro D (1991). Increased concentrations of tumor necrosis factor in ‘cachectic’ patients with severe heart failure.. Br Heart J.

[33] Torre-Amione G, Kapadia S, Lee J, Bies RD, Lebovitz R, Mann DL (1995). Expression and functional significance of tumor necrosis factor receptors in human myocardium.. Circulation.

[34] Dutka DP, Elborn,, Delamere F, Shale DJ, Morris GK (1993). Tumour necrosis factor α in severe congestive cardiac failure.. Br Heart J.

[35] Tashiro H, Shimokawa H, Yamamoto K (1995). Monocyte-related cytokines in acute myocardial infraction.. Am Heart J.

